# CXCL10 increases in human skeletal muscle following damage but is not necessary for muscle regeneration

**DOI:** 10.14814/phy2.13689

**Published:** 2018-04-25

**Authors:** Michael R. Deyhle, Paul S. Hafen, Jacob Parmley, Coray N. Preece, Marissa Robison, Jacob R. Sorensen, Blake Jackson, Dennis L. Eggett, Chad R. Hancock, Robert D. Hyldahl

**Affiliations:** ^1^ Department of Exercise Sciences Brigham Young University Provo Utah; ^2^ Department of Statistics Brigham Young University Provo Utah; ^3^ Department of Nutrition Dietetics & Food Science Brigham Young University Provo Utah

**Keywords:** chemokine, CXCR3 ligands, inflammation, IP‐10, T cell

## Abstract

CXCL10 is a chemokine for activated and memory T cells with many important immunological functions. We recently found that CXCL10 is upregulated in human muscle following contraction‐induced damage. No information is available on the role of CXCL10 in the context of muscle damage or repair. In this study, we confirm that CXCL10 is elevated in human muscle at 2 and 3 days following damage and perform cell culture and animal studies to examine the role of CXCL10 in muscle repair. CXCL10 did not impact proliferation rates of human primary myoblasts but it did promote myogenic differentiation in vitro, suggesting a possible direct impact on muscle regeneration. To test if CXCL10 was dispensable for effective muscle regeneration in vivo*,* we measured functional and histological markers of muscle repair out to 14 days postmuscle injury caused by a myotoxin in wild‐type (WT) mice and CXCL10 knockout (KO) mice. Between genotypes, no significant differences were found in loss or restoration of in situ muscle force, cross‐sectional area of newly formed myofibers, or the number of embryonic myosin heavy chain‐positive myofibers. In addition, KO animals were not deficient in T‐cell accumulation in the damaged muscle following injury. Gene expression of the other two ligands (CXCL9 and 11) that bind to the same receptor as CXCL10 were also elevated in the damaged muscle of KO mice. Thus, other ligands may have compensated for the lack of CXCL10 in the KO mice. We conclude that CXCL10 is not necessary for effective muscle regeneration.

## Introduction

Skeletal muscle is a highly specialized postmitotic tissue that is particularly susceptible to injury. Accordingly, it possesses a robust repair mechanism that appears to be dependent on the timely infiltration of specific subsets of immune cells (Peake et al. 2017; Tidball 2017). Studies have demonstrated the significance of neutrophils, monocytes, macrophages, mast cells, and eosinophils, in the muscle repair process (Heredia et al. 2013; Peake et al. 2017; Tidball 2017). In addition to myeloid cells, T lymphocytes have more recently emerged as valuable contributors to the repair process following severe muscle injury (Burzyn et al. 2013; Fu et al. 2015; Kuswanto et al. 2016; Zhang et al. 2014). Secreted signaling molecules, such as cytokines, chemokines, and growth factors, are often central to the muscle repairing functions of immune cells. Among those known to play important roles in muscle repair include CCL2 (Sun et al. 2009), CXCL16 (Zhang et al. 2009), TNF (Fu et al. 2015; Warren et al. 2002), IL‐4 (Heredia et al. 2013), IFN‐*γ* (Cheng et al. 2008; Fu et al. 2015), IL‐33 (Kuswanto et al. 2016), amphiregulin (Burzyn et al. 2013) and IGF‐1 (Lu et al. 2011b).

To gain a better understanding of how cytokine/chemokine signaling influences repair of human skeletal muscle, we recently used high‐throughput multiplexing analyses to assess cytokine expression following contraction‐induced muscle damage in humans (Deyhle et al. 2015). Surprisingly, among the 29 cytokines measured, only two significantly increased following damage. One of the upregulated cytokines was the chemokine CCL2. The role of CCL2 in muscle repair has been well established. CCL2 is required for effective muscle regeneration by recruiting monocytes to injured muscles (Lu et al. 2011a; Sun et al. 2009) and influencing their phenotype (Arnold et al. 2015). The other upregulated cytokine was the chemokine C‐X‐C motif ligand 10 (CXCL10), also known as interferon‐*γ*‐inducible protein 10 (IP‐10). Unlike CCL2, information on the role of CXCL10 in the context of muscle damage and repair is scarce. CXCL10 has been implicated in pathological muscle conditions such as inflammatory myopathies (Crescioli et al. 2012), but to our knowledge there has been no investigation into the role of CXCL10 in the context of muscle damage and repair in otherwise healthy muscle. CXCL10 binds to the G‐protein‐coupled receptor CXCR3 to elicit various responses including the directed migration of T cells (Dufour et al. 2002; Hoerning et al. 2011; Taub et al. 1993), T‐cell adhesion to endothelial cells (Taub et al. 1993), and T‐cell effector polarization (Zohar et al. 2014). As with most chemokine/receptor axes, additional ligands (CXCL9 and CXCL11) can also engage CXCR3. While many of the functions exerted by each ligand are redundant or collaborative, even antagonistic functions have been reported among the CXCR3 ligands (Groom and Luster 2011; Zohar et al. 2014). Due to the complexity in signaling through CXCR3, studies investigating the function of any one ligand should account for the activity of the others.

Given its robust expression in human muscle following damage, CXCL10 might carry out important functions needed for muscle repair. A possible mechanism by which CXCL10 could support muscle repair is by recruiting T cells, which are needed for proper muscle regeneration (Burzyn et al. 2013; Fu et al. 2015; Kuswanto et al. 2016; Zhang et al. 2014). CXCL10 is known to promote directed T‐cell migration (Harris et al. 2012; Khan et al. 2000). In support of this supposition, we previously found that muscle CXCL10 content significantly correlated with the numbers of intramuscular CD8+ T cells in humans following contraction‐induced damage (Deyhle et al. 2015). Thus, we hypothesize that CXCL10 is needed for effective muscle regeneration. Herein, we report a more thorough time course of CXCL10 expression following contraction‐induced damage in humans, test the effect of CXCL10 treatment on human primary myoblasts, and also test the importance of CXCL10 in muscle functional restoration, tissue regeneration, and T‐cell accumulation following toxin‐induced injury.

## Methods

### Human Studies

#### Subjects

A total of 10 healthy individuals volunteered to participate in this study (6 Men; 21.5 ± 1.2 year., 83 ± 12.6 kg, 177 ± 3.6 cm and 4 women; 23 ± 3 years, 64.6 ± 13.4 kg, 168.4 ± 9.5 cm). The subjects were not participating in any resistance training or regularly scheduled physical exercise, and thus were considered as physically inactive. Subjects were informed of all the study procedures as well as potential risks associated with participating prior to signing an informed consent document. The Brigham Young University Institutional Review Board approved all study procedures prior to our seeking subjects.

#### Muscle damaging contractions and function assessment

We used established methods to induce muscle damage and measure muscle strength (Deyhle et al. 2016). To cause a contraction‐induced injury, subjects performed 30 sets of 10 maximal‐effort lengthening (60^o^/sec) contractions of the knee extensors on an isokinetic dynamometer (Biodex Medical Systems, Shirley, NY). To provide an index as to the degree of muscle damage caused by the lengthening contractions, isometric muscle strength was measured on the same dynamometer before the damaging contractions as well as immediately, 24, and 72 h after. To measure isometric strength, the subjects performed three 5‐sec‐long maximal voluntary contractions of the knee extensors with the knee fixed at 70°. Both the lengthening contractions and the strength tests were done unilaterally on the same randomly selected limb.

#### Muscle biopsy procedure

Muscle samples were obtained from the vastus lateralis muscle at the four following time points: 1 day before, 3, 24, and 72 h after the lengthening contractions. The baseline biopsy was obtained from the nonexercised leg, while the postexercise samples were obtained from the exercised leg. To perform the biopsy, a sterile field was established on and around the limb then a 2% Lidocaine solution was injected into the leg as a local anesthetic. A small incision was made through the skin and fascia approximately 15 cm proximal to the insertion of the vastus lateralis. A Bergstörm biopsy needle was inserted into the muscle and the sample was extracted with a suction‐assisted maneuver. The muscle sample was cleared of fatty and connective tissue and then snap frozen in liquid nitrogen. The sample was then stored at −80°C. Subsequent biopsies obtained from the same limb (e.g., 24 h and 72 h biopsies) were taken 3–5 cm proximal to the previous biopsy incision, and the needle was angled away from the previous site. This practice has been shown to minimize artifact caused by repeated biopsies (Van Thienen et al. 2014).

#### CXCR3 ligand magnetic bead assay

Frozen tissue samples were weighed (41.8 ± 18 mg) and homogenized in lysis buffer (cat# 43‐040 from Millipore) at a ratio of 9 *μ*L per mg tissue. HaltTM protease and phosphatase inhibitor cocktail (100X) (cat# 78440, Thermo Fischer Scientific; Salt Lake City, UT) was also added to the homogenate. Tissue was homogenized then centrifuged at 10,000*g*, 4°C for 10 min. The supernatant was analyzed in triplicate for total protein concentrations with the BCA Protein Assay Kit (product# 23227, Thermo Scientific). Supernatant was stored at −80°C. CXCL9, CXCL10, and CXCL11 protein concentrations were measured using a MAGPIX multiplexing platform (Luminex xMAP Technology, San Diego, CA). Analysis was performed using reagents supplied by Millipore according to the manufacturer's recommendations. Antibody‐conjugated magnetic beads were incubated with 25 *μ*g of total protein from tissue homogenate overnight at 4°C on a plate shaker. Bead‐complexes were then washed and incubated in biotinylated detection antibodies on a plate shaker at room temperature for 60 min. Next, the samples were incubated in phycoerythrin‐conjugated streptavidin for 30 min at room temperature. Bead‐complexes were resuspended in assay buffer and mixed on a plate shaker for 5 min, and then analyzed on a MAGPIX multiplex fluorescent imager. Mean fluorescent intensities were recorded and used for data analysis. Data were analyzed using Milliplex Analyst 5.1 software (Millipore Corporation, Billerica, MA).

#### Human myoblast isolation

Using a published method (Agley et al. 2015), human primary myoblasts were isolated from skeletal muscle biopsy samples obtained from a 31‐year‐old healthy Caucasian male. In brief, the muscle biopsy samples were weighed, cut, and enzymatically digested for 60 min (2 mg/mL Collagenase D, 2 mg/mL Dispase II). Following enzymatic digestion the cells were strained (100 *μ*m) and resuspended in growth medium (Dulbecco's modified eagle medium with 20% fetal bovine serum and 1% penicillin/streptomycin). Cells were incubated at 37°C with 5% CO_2_ for 7 days. Growth medium was replaced every 48 h. Following incubation, myoblasts were isolated through a magnetic separating column using anti‐CD56 immunomagnetic beads (Miltenyi Biotec; Bergish Gladbach, Germany).

#### Proliferation assay

Human primary myoblasts were suspended in either growth medium only (*n* = 16 wells), or in growth medium plus recombinant Human CXCL10 (R&D Systems; Minneapolis, MN) at concentrations of 10 pg/mL (*n* = 10), 100 pg/mL (*n* = 12), or 10 ng/mL (*n* = 12). Cells were plated on matrigel at a density of approximately 1000 cells/cm^2^, incubated at 37°C/5% CO_2_, and allowed to attach overnight. These concentrations were chosen based on the CXCL10 concentrations that we have observed in muscle tissue previously (Deyhle et al. 2015; Hyldahl et al. 2014) and in this study. 5‐Ethynyl‐2′‐deoxyuridine (EdU) was added to each well to mark proliferating cells. Cells were harvested at 6 and 48 h and prepared in accordance with Click‐iT^®^ EdU Alexa Fluor^®^ 488 Imaging Kit protocols (Invitrogen^TM^). DAPI (4′,6‐diamidino‐2‐phenylindole) was used to measure total cell count. Proliferation was measured by the ratio of EdU‐positive nuclei to the total nuclei (Edu^+^ Cells/Total Cell Count).

#### Differentiation assay

Isolated myoblasts were suspended in growth medium, plated on matrigel at a density of approximately 4000 cells/cm^2^ and incubated at 37°C with 5% CO_2_ until reaching ~70% confluence. The medium was then changed in order to induce differentiation of myoblasts into myotubes (Dulbecco's modified eagle medium with 2% horse serum and 1% penicillin/streptomycin). The wells were randomly treated with either differentiation medium only (*n* = 8), or with differentiation medium plus CXCL10 in concentrations of 10 pg/mL (*n* = 6), 100 pg/mL (*n* = 6), or 10 ng/mL (*n* = 6). The cells were allowed to differentiate and were harvested after 24 h. Differentiation was determined by quantifying myotube area via myosin heavy chain (MyHC) expression. Following incubation in differentiation medium for 24 h, cells were rinsed in PBS and fixed in 3% paraformaldehyde (PFA). Cells were then permeabilized in 0.5% Triton® X‐100 for 3 min followed by another 2 min of fixation in 3% PFA. Cells were rinsed twice in PBS with 20% tween (PBST) and then blocked in 3% bovine serum albumin (BSA) and 5% FBS for 30 min at room temperature. Cells were rinsed in PBS and incubated in the MyHC primary antibody MF 20‐S mouse anti‐human myosin (Developmental Studies Hybridoma Bank, diluted 1:100 in PBS) in the dark at room temperature for 60 min. After incubation in the primary antibody, the cells were rinsed three times in PBST, and then incubated with DAPI and the secondary antibody Alexa Fluor® 488‐conjugated AffiniPure Goat Anti‐Mouse IgG (H + L) (Life Technologies, diluted 1:100 in PBS) for 60 min at room temperature in the dark.

### Animal study

#### Mice

The BYU IACUC approved the experimental protocol prior to obtaining the study animals. Ten‐week‐old male wild‐type (WT, c57bl/6J, 23.8 ± 1.9 g) and CXCL10 knockout (KO, B6.129S4‐Cxcl10^tm1Adl^/J, 24.2 ± 1.6 g) mice were purchased from the Jackson Laboratory (Bar Harbor, ME). Mice were housed in pairs on a standard 12‐h light and dark cycle and were left unperturbed for 5 days after arrival at the laboratory prior to starting any experimental intervention.

#### Muscle injury

Buprenorphine (0.05 mg/kg) was administered by intraperitoneal injection as a proactive analgesic 1 h prior to the muscle injury. To induce the muscle injury, mice were anesthetized using inhaled isoflurane (2%) in pure oxygen. Sufficient depth of anesthesia was confirmed by the absence of toe‐pinch reflex. Fur was removed from the anterior part of the lower limb using clippers. The tibialis anterior of one limb was injected with 25 *μ*L of a 10 mmol/L cardiotoxin (CTX) solution (Sigma‐Aldrich; St. Louis, MO). The opposite limb was injected with an equal volume of sterile isotonic saline to serve as a sham control. The injection procedure was done as follows; the needle was inserted at the distal end of the tibialis anterior and was advanced longitudinally through the middle of the muscle to the proximal end. The needle was then withdrawn as the contents of the syringe were dispensed into the muscle. Care was taken to distribute the injection as evenly as possible throughout the muscle. After the injections, the mouse was monitored until recovery in a heated home cage.

#### In situ muscle function assessment

At 2, 7, and 14 days postinjury (DPI) maximal isometric tetanic contraction force of the tibialis anterior muscle was measured in situ using the Aurora Scientific 1300A muscle test system. The mouse was anesthetized using 2–2.5% isoflurane vaporized in pure oxygen. Once anesthetized, the body mass was measured. The absence of pedal reflexes indicated the sufficient depth of the anesthesia. The skin of the lower limb was removed to expose the tibialis anterior. The distal tendon of the tibialis anterior was cut and secured to the force transducer with surgical silk suture. The femur was then secured to the heated surgical plate between two pins. The sciatic nerve was exposed and tied off with thread to allow for direct electrode‐to‐nerve stimulation to elicit contractions. Both limbs were prepared for the muscle function assessment as described above. Ringers buffer‐soaked cotton patches were placed over the exposed limbs to prevent drying. To assess isometric tetanic force production and fatigue curves, contractions were elicited every 2 sec for a total of 5 min (150 contractions total) by stimulating the nerve with a Grass S88X stimulator (100 msec trains of 0.05‐msec square waves at a frequency of 150 Hz). The minimum voltage required to elicit maximal contraction force was used on a case‐by‐case basis (between 2 and 7 v). This procedure was adapted from previous work (Hancock et al. 2005; Thomson et al. 2010) and a published method (Hakim et al. 2013). Three to five mice per genotype per time point were analyzed.

#### Tissue sampling and preparation

At the conclusion of the muscle function assessment, the mice were killed by exsanguination (using vena cava blood draw) followed by cervical dislocation while deeply anesthetized. Both tibialis anterior muscles were then carefully excised, blotted on a filter paper, weighed, and frozen in liquid nitrogen‐cooled isopentane for later analysis. Cross‐sections of 8 *μ*m thick were cut from the midbelly of the muscle samples using a cryostat (−27°C). Sections were mounted on glass slides and stored at −80°C for future use. After obtaining muscle sections for histology, the remainder of the muscle sample (32 ± 1.2 mg) was mechanically disrupted and homogenized using a plastic pestle in a 1.5‐mL microcentrifuge tube with 900 *μ*L of QIAzol Lysis Reagent (QIAGEN, Hilden, Germany) and RNA was purified using a RNeasy Plus Universal kit (QIAGEN) according to the protocol provided in the kit.

#### Muscle histology and immunohistochemistry

To observe general muscle architecture and measure muscle fiber cross‐sectional area (CSA), samples were stained using hemotoxylin and eosin (H&E). The stained samples were covered with glass coverslips with Canada balsam mounting medium (Sigma‐Aldrich). To quantify the number of fibers undergoing regeneration, samples were stained with an anti‐embryonic myosin heavy chain (eMyHC) antibody. Samples were removed from the freezer and allowed to dry for 10 min. Then, they were fixed in 2% paraformaldehyde for 5 min and were washed in PBS. Next, they were incubated in a blocking solution (5% goat serum in PBS) for 30 min and washed again. The samples were then incubated in a humidified chamber overnight (9–10 h at −20°C) with the primary antibodies (anti‐eMyHC and anti‐dystrophin diluted in a blocking cocktail at 1:25 and 1:100, respectively). Samples were then washed and incubated in the secondary antibodies (Cy3 and AlexaFluor 488) diluted in a blocking cocktail at 1:100 and 1:100, respectively, and DAPI at room temperature for 30 min. Stained slides were then washed three times for 5 min each in PBS, and then mounted using glycerol as a mounting medium. Primary antibodies used were F1.652‐s (anti‐eMyHC, Developmental Studies Hybridoma Bank, Iowa City, IA) and Mandra15(3G11)‐s (anti‐dystrophin, Developmental Studies Hybridoma Bank). Secondary antibodies used were AlexaFluor 488 goat anti‐mouse IgG (H + L) 115‐545‐003 (Jackson ImmunoResearch Laboratories West Grove, PA) and Cy3 anti‐rabbit IgG (H + L) 111‐165‐003 (Jackson). To observe the concentration of T‐cells bearing the CXCL10 receptor (CXCR3) present in the WT and KO muscle over the course of the repair process, samples were costained with anti‐CD3 and anti‐CXCR3 antibodies. Slides were removed from the freezer and allowed to thaw and dry for 10 min. Then, they were fixed in 2% paraformaldehyde for 10 min. After fixation, samples were washed in PBS, and then blocked using 5% goat serum and a drop of M.O.M. IgG blocking reagent (MKB‐2213, Vector Laboratories; Burlingame, CA) solution with PBS for 60 min. The blocking solution was removed and the samples were then incubated in primary antibodies overnight at 4°C (anti‐CD3 diluted 1:50, anti‐CXCR3 diluted 1:50). On the following day, samples were washed (4 × 5 min), and incubated in secondary antibodies diluted 1:100 in PBS for 30 min at 37°C. A quantity of 1 *μ*L of DAPI was added to the secondary antibody solution to visualize nuclei. Following incubation, samples were washed in PBS, and dipped in distilled water. The stained slides were then mounted with Fluoroshield histology mounting medium (Sigma‐Aldrich). Primary antibodies used were rat anti‐mouse CD3 (MAB4841) from (R&D System; Minneapolis, MN) and rabbit anti‐mouse CXCR3 bs‐2209R (Bioss Antibodies; Woburn, MA). Secondary antibodies used were Cy3‐conjugated goat anti‐rat IgG (112‐165‐044) from Jackson ImmunoResearch Laboratories (West Grove, PA, USA) and Alexa Fluor^®^ 488‐conjugated goat anti‐rabbit IgG from Jackson ImmunoResearch Laboratories. The validity of each immunostaining method was verified using secondary antibody‐only controls.

#### Real‐time PCR

Total RNA concentration (absorbance at 260 nm) and purity (260/280 ratio) of the RNA sample were measured using a spectrophotometer (Nanodrop 2000, ThermoFisher Scientific; Salt Lake City, UT). RNA concentration was 420 ± 349 ng/mL and the 260/280 ratio averaged 2.03 ± 0.05. A quantity of 20 ng of RNA from each sample was reverse transcribed using a iScript™ Select cDNA Synthesis Kit (Bio‐Rad Laboratories; Hercules, CA). cDNA concentration was measured using a spectrophotometer (Nanodrop 2000). Real‐time PCR was run using the CFX connect™ Real‐Time System with the following Primetime primers CXCL9 (assay ID: Mm.PT.58.13098261), CXCL10 (assay ID: Mm.PT.58.43575827), and CXCL11 (assay ID: Mm.PT.58.42838989) (Integrated DNA Technologies; Coralville, IA). We did not measure CXCL11 in the WT samples because c57bl/6 mice have a neutralizing mutation in the CXCL11 gene (Groom and Luster 2011; Sierro et al. 2007; Zohar et al. 2014); thus, CXCL11 mRNA in these animals would be irrelevant because it fails to encode an effectual protein. Because CXCL10 KO mice were developed with the use of J1 embryonic stem cells (which harbor an intact *cxcl11* gene), the KO animals have an intact *cxcl11* gene (Dufour et al. 2002; Groom and Luster 2011). Because the KO mice, unlike their background strain, are expected to express a functional CXCL11 gene, we did measure CXCL11 in the KO animals. The thermocycling protocol consisted of one cycle for 2 min at 95°C, then 40 cycles for amplification (5 sec at 95°C followed by 30 sec at 60°C). After amplification cycles, a melt curve step was done to verify product purity of target amplification. Fluorescence (iTaq universal SYBR Green Supermix, Bio‐Rad Laboratories) was measured after each thermo cycle. All samples were run in triplicate on 96 well plates. No‐template controls were run for each primer. CTX injury results in complete destruction of muscle fibers thus, it is difficult to find a stable housekeeping gene in the face of a CTX injury. Thus, gene expression was calculated by 2^−CT^ normalized to the concentration of cDNA in the PCR the reaction, as others have done (Swiderski et al. 2016; van Poel et al. 2011).

#### Microscopy

All fluorescent micrographs were obtained using an Olympus IX73 microscope and Olympus XM10 camera. An Olympus SC50 color camera was used for obtaining images of H&E‐stained samples. When imaging the CTX‐injected muscle samples, the whole damaged area was imaged with sequential nonoverlapping images. Several randomly selected nonoverlapping images were taken when imaging the saline‐injected (sham) limb. The 10× objective was used to capture all fluorescent images and the 20× objective was used for H&E images.

#### Micrograph quantification

All micrograph quantification was done by investigators who were blind to the experimental condition of the sample using Olympus cellSens™ microscope imaging software. For the cell culture proliferation and differentiation assays, five random, nonoverlapping images from each treatment condition were manually quantified using the imaging software. Proliferated cells were identified by the presence of EdU‐positive nucleus (localized with DAPI). Differentiated myotubes were identified by the presence of MyHC and the total MyHC‐positive area was measured. The fusion index was quantified by counting the total number of nuclei and the percentage of the total present inside of myotubes. Only myotubes containing three or more nuclei were counted. CSA was measured from the H&E images by manually outlining individual muscle fibers. Only the regenerating fibers (centrally nucleated fibers) were measured in the CTX‐injected muscle, while only normal, undamaged fibers were measured in the sham‐injected muscle. An average of 252 ± 126 centrally nucleated fibers and 102 ± 30 normal fibers per mouse were measured for the analysis. No centrally nucleated fibers were observed at the 2 DPI time point. Therefore, 2 DPI was not included in the analysis for CSA. The number of muscle fibers expressing eMyHC was counted at 7 and 14 DPI. The entire damaged area of the muscle stained positive for eMyHC at the 2 DPI time point, and it was not possible to distinguish among individual fibers because the damaged muscle fibers at this time did not stain positive for the membrane‐associated protein dystrophin. Therefore, 2 DPI was not included in the analysis. Muscle fibers that stained positive for eMyHC (Alexa Fluor® 488/green) surrounded by a dystrophin‐positive (Cy3/red) membrane were counted as eMyHC‐positive muscle fibers. The number of CD3^+^ (Cy3/red) and CXCR^+^ (Alexa Fluor® 488/green) cells were identified by the bright punctate stain of their respective colors surrounding a DAPI+ nucleus. Brightly stained spots without nuclear colocalization were not counted as cells.

#### Statistical analysis

For the human study, data were analyzed using a two‐way mixed models analysis. Fixed effects were sex, time, and sex‐by‐time interaction. A subject identifier variable was included as a random effect and was nested with sex and crossed with time. Human CXCL10, CXCL9, and CXCL11 protein data were analyzed on the log_10_ scale due to nonhomogenous variance (assessed by examining the plot of residuals). For the cell culture study, proliferation data were analyzed using a two‐way analysis of variance for effect of time (6 h, 24 h) and CXCL10 treatment (0 pg/mL, 10 pg/mL, 100 pg/mL, and 10 ng/mL). Differentiation data were analyzed using an analysis of variance for the effect of CXCL10 treatment (control, 10 pg/mL, 100 pg/mL, and 10 ng/mL) on differentiation, measured by area positive for MyHC. The log_10_ of CXCL10 on the MyHC‐positive area was analyzed to investigate a log [dose] response relationship. For the mouse study, data were analyzed using two‐ or three‐way mixed models analysis where appropriate. For three‐way analyses, fixed effects were genotype (KO or WT), DPI (2, 7 and 14 days), and treatment (CTX or sham). Body mass was also included when appropriate to account for variance due to this variable. Interactions were included in the model where appropriate. A mouse identifier variable was included as a random effect. Genotype and DPI were nested in the mouse identifier variable, while treatment was crossed with it. Two‐way analyses were used when it was not appropriate to include the treatment variable. This was the case when the dependent variable of an analysis was expressed as a percentage of the values on the sham muscle. Tukey's post hoc tests were used for pairwise comparisons when the *F*‐statistic of a main effect revealed a significant *P*‐value (<0.05). Data are presented as mean ± standard deviation (SD). All data were analyzed using JMP^®^ Pro version 12.0.1 (SAS institute Inc., Cary, NC). Figures were made using Prism 7 for Mac OS version 7c. (GraphPad software, La Jolla, CA).

## Results

### Human studies

#### Maximal isometric torque loss

**Figure 1:**
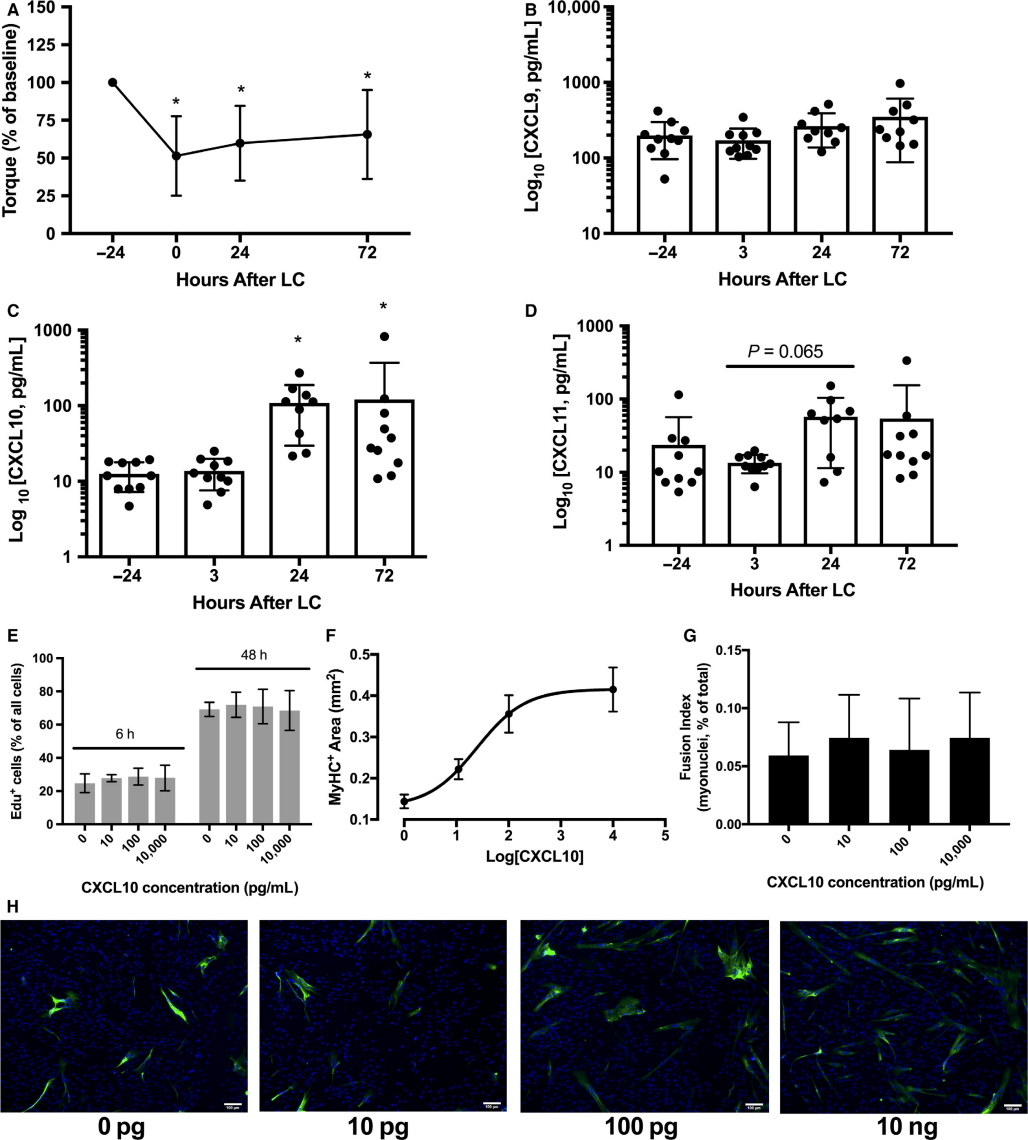
(A) Maximal voluntary isometric knee extensor force was significantly reduced relative to baseline immediately after as well as 24, 48, and 72 h after a bout of 300 maximal‐effort lengthening contractions (LC). (B) Muscle CXCL9 content remained unchanged out to 72 h following LC. (C) Muscle CXCL10 concentration was elevated at 24 h and 72 h after LC. (D) Muscle CXCL11 concentration did not change significantly out to 72 h after lengthening contractions. (E) CXCL10 did not impact proliferation rates of human primary myoblasts at 6 or 48 h. (F) Log_10_ [dose] response of CXCL10 on human primary myoblast differentiation as measured by MyHC (myosin heavy chain)‐positive area. (G) The percentage of total nuclei present inside of myotubes (fusion index) was not different among the different CXCL10 treatment conditions. (H) Representative images of cells treated with four different concentrations of CXCL10. Data are mean ± SD. * indicates significantly different than −24 h (*P *< 0.05).

A reduction in maximal isometric torque production is a reliable and valid indicator of the degree of muscle damage (Paulsen et al. 2012). To verify that the lengthening contraction protocol resulted in damage to the knee extensors, maximal voluntary isometric torque was measured before and out to 72 h following lengthening contractions (Fig. 1A). Relative to preexercise values, maximal isometric torque was significantly reduced immediately postexercise (49 ± 26%, *P* < 0.0001), 24 h postexercise (40 ± 25%, *P* = 0.0005), and 72 h postexercise (34 ± 29%, *P* = 0.0024). Based on the magnitude of strength loss and rate of strength recovery observed here, the degree of contraction‐induced muscle injury is categorized as moderate (Paulsen et al. 2012).

#### CXCR3 ligand expression

We have previously reported elevated levels of CXCL10 protein in damaged muscle 24 h after a single bout (Hyldahl et al. 2014), and at 72 h after a repeated bout (Deyhle et al. 2015) of lengthening contractions. To confirm this finding and more rigorously characterize the time course of intramuscular CXCR3 ligand fluctuation following damage, we measured CXCL9, CXCL10, and CXCL11 at 3, 24, and 72 h following contraction‐induced damage (Fig. 1B, C, and D). Relative to predamage values (13 ± 5 pg/mL), CXCL10 was not elevated at 3 h (13.67 ± 6 pg/mL, *P* > 0.05) but was significantly increased at 24 h (109 ± 79 pg/mL, *P* = 0.0003) and 72 h postdamage (120 ± 249 pg/mL, *P* = 0.01 Fig. 1C). The chemokines CXCL9 and CXCL11 bind to a common receptor (CXCR3) with CXCL10. Because CXCL10 is elevated in muscle following damaging contractions, we sought to quantify the CXCL9 and CXCL11 content in the muscle samples. CXCL9 did not change significantly (*P* = 0.06, Fig. 1B) following muscle damage (preexercise: 198 ± 101 pg/mL, 3 h: 171 ± 73 pg/mL, 24 h: 263 ± 106, 72 h: 350 ± 262 pg/mL). Men had higher levels of CXCL9 than women (Sex, *P* = 0.047). For CXLC11, there was a significant main effect for Time (*P* = 0.049). However, no significant differences were found after correcting for pairwise comparisons among the individual time points (pre exercise: 24 ± 33 pg/mL, 3 h: 13 ± 4, 24 h: 263 ± 125 pg/mL, 72 h: 54 ± 100 pg/mL, Fig. 1D).

#### Proliferation and differentiation of human primary myoblasts

Proliferation and differentiation of muscle stem cells (satellite cells) are indispensible in the process of effective muscle regeneration following severe injury to skeletal muscle (McCarthy et al. 2011) and many cytokines and other paracrine signaling mediators provide important regulatory control over muscle stem cell activity (Tidball 2017). Among the three ligands that bind CXCR3, CXCL10 was the only one that increased significantly following contraction‐induced muscle damage (Fig. 1C). Therefore, we treated human primary myoblasts with CXCL10 to test the effect of CXCL10 on proliferation or differentiation.

After 6 h, the percentage of cells positive for EdU (proliferated cells) was not statistically different (*P* = 0.5410) between the control (24.7 ± 5.7%) and treatment groups exposed to CXCL10 concentrations of 10 pg/mL (27.8 ± 2.1%), 100 pg/mL (28.7 ± 5.1%), and 10 ng/mL (27.9 ± 7.7%). A larger percentage of cells had proliferated over the course of 48 h, yet the extent of proliferation remained similar (*P* = 0.8960) between the control (69.1 ± 4.3%) and treatment groups of 10 pg/mL (71.9 ± 7.6%), 100 pg/mL (70.9 ± 10.4%), and 10 ng/mL (68.5 ± 12.0%) (Fig. 1E). At 24 h following the initiation of differentiation, there was a significant effect of CXCL10 treatment on myotube differentiation as measured by the amount of MyHC‐positive area (*P* < 0.0001, Fig. 1F). MyHC‐positive area was lowest in the control cells (0.144 ± 0.048 mm^2^) and increased at concentrations of 10 pg/mL (0.221 ± 0.060 mm^2^), 100 pg/mL (0.356 ± 0.111 mm^2^), and 10 ng/mL (0.415 ± 0.132 mm^2^; Fig. 1F). We observed a significant positive log_10_(dose) response relationship (*r* = 0.761, *P* < 0.0001) between log[CXCL10] and MyHC‐positive area (mm^2^) (Fig. 1F). CXCL10 treatment had no effect on the fusion index (Fig. 1G). Figure 1H shows representative images from each treatment condition of the differentiation assay.

### Animal study

Because CXCL10 is elevated in human muscle following contraction‐induced damage (Deyhle et al. 2015; Hyldahl et al. 2014) and CXCL10 treatment significantly increased the differentiation activity of human primary myoblasts (Fig. 1F), we designed a study using KO mice to test whether CXCL10 was dispensable for muscle regeneration in vivo.

#### Muscle function

**Figure 2:**
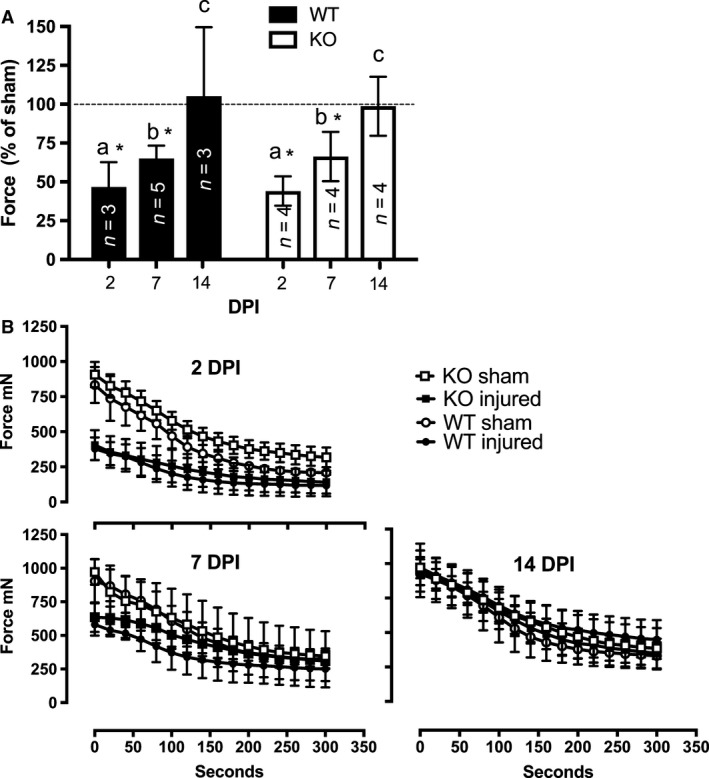
(A) Maximal isometric in situ force of injured (cardotoxin‐injected) muscle expressed as a percentage of sham (saline‐injected) muscle force at 2, 7, and 14 days postinjury (DPI) in wild‐type (WT) and CXCL10 knockout (KO) mice. Data not connected by the same letter (a, b, or c) are statistically different (*P *< 0.05). * indicates significant difference from sham (dotted line). (B) Maximal isometric tetanic contractions were elicited in situ at a rate of 0.5 Hz for 300 sec. The first and every 10 contractions thereafter are plotted for both injured and sham muscles of both genotypes. Injured and sham muscle force curves were divergent at 2 and 7 DPI in both genotypes. Injured and sham muscle force curves were similar by 14 DPI in both genotypes. Data are mean ± SD. *n* = 3–6 per experimental condition.

To test whether the KO mice lost more force postinjury or took longer to recover force production postinjury than the WT mice, we measured the force generated during peak tetanic contractions of the tibialis anterior in situ of both the injured and uninjured limbs (Fig. 2). Peak tetanic force (Fig. 2A) of the injured limb was significantly reduced relative to the sham limb at 2 (WT: 46 ± 16%, KO: 44 ± 9.4%, *P* = 0.0001) and 7 DPI (WT: 65 ± 8%, KO, 66 ± 16%, *P* = 0.0003) and was restored to similar levels as the sham limb at 14 DPI (WT: 105 ± 44%, KO 99 ± 19% *P* = 1). No significant differences were found between genotypes (genotype: *P* = 0.3, genotype × treatment × DPI: *P* = 0.973) (Fig. 2A). Specific force (mN/mg of TA) was significantly reduced (*P* <0.0001) in the injured muscles (CTX‐injected) compared to noninjured muscles (sham) at 2 DPI in both WT (7.8 ± 1.3 vs.17.5 ± 2.7) and KO mice (9.1 ± 2.6 vs. 20 ± 3.8). Specific muscle force remained lower in the injured muscles compared to sham muscles at 7 DPI (*P* = 0.02) in both WT (16.3 ± 2.9 vs. 21.2 ± 3.6) and KO mice (15.1 ± 2.5 vs. 20.7 ± 1.1). By 14 DPI, specific force was not different between injured and sham muscles (*P* = 0.94) in both WT (18.4 ± 1.2 vs. 19.3 ± 2.7) and KO mice (19.5 ± 2.6 ± 20.8 ± 2.3).

Figure 2B shows the absolute values in peak muscle force over the course of the contraction bout. At 2 and 7 DPI, the force curves were divergent between injured and sham muscles but by 14 DPI the force curves between injured and sham limbs were indistinguishable (Fig. 2B). No significant differences were found between genotypes for the degree or rate of fatigue, maximum force derivative, or half‐relaxation times (all *P* values > 0.05, data not shown).

#### Muscle mass

**Figure 3:**
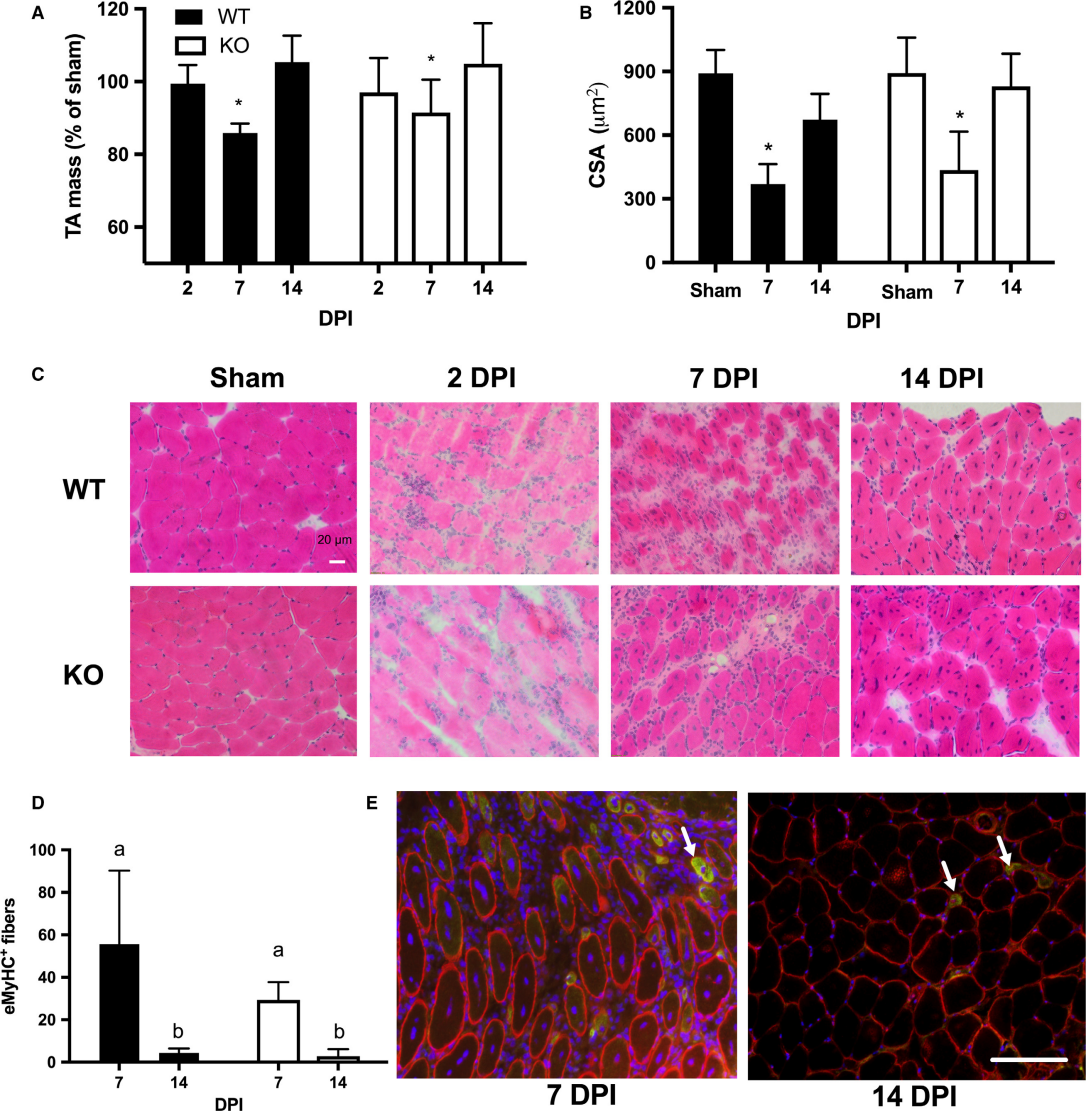
(A) Cardiotoxin‐injected (injured) tibialis anterior (TA) muscle mass relative to sham (saline‐injected) muscle mass. Injured muscles were significantly lighter at 7 days postinjury (DPI) in both wild‐type (WT) and CXCL10 knockout (KO) mice. (B) Regenerating (central nucleated) muscle fiber cross‐sectional area (CSA) was significantly reduced in both genotypes at 7 DPI (*P < 0.05) but was recovered similar to muscle fibers of sham muscles by 14 DPI. (C) Representative H&E images from sham and injured muscles. (D) The number of muscle fibers expressing embryonic myosin heavy chain (eMyHC) was greater at 7 DPI in both genotypes. Bars not sharing the same letter (a or b) are significantly different (*P* < 0.05). (E) Representative images of eMyHC stain at 7 and 14 DPI in WT animals. Red = dystrophin, Green = eMyHC, Blue = DNA. Arrowheads denote examples of eMyHC‐positive muscle fibers. Data are mean ± SD. *n* = 4–6 per experimental condition. Scale bar is 100 *μ*m.

As a general indicator of the degree of muscle damage and repair, we examined injured muscle mass expressed as a percentage of the sham muscle (Fig. 3A). The clearance of damaged and necrotic tissue from the muscle should be indicated by the relative loss of muscle mass of the damaged muscle. As the damaged tissue is replaced and regenerated, the muscle mass is expected to be restored back to noninjured values. The injured muscles were significantly smaller at 7 DPI (WT: 85 ± 5%, CXCL10: 91 ± 9%) compared to 14 DPI (WT: 105 ± 7%, 104 ± 11%), yet no significant differences were observed between genotypes (genotype *P* = 0.7, genotype × DPI *P* > 0.6).

#### Cross‐sectional area

To test whether the absence of CXCL10 resulted in delayed muscle fiber growth, we compared the CSA of regenerating (centrally nucleated) muscle fibers in the CTX‐injected muscles at 7 and 14 DPI to undamaged fibers of the sham muscles at 7 and 14 DPI (Fig. 3B). Representative images from are shown in Figure 3C. Sham muscles at 7 and 14 DPI were grouped together for the analysis. The mean CSA in the damaged muscles was significantly reduced at 7 DPI in both genotypes (WT: 370 ± 94 *μ*m^2^, KO: 436 ± 181 *μ*m^2^, *P* < 0.0001) compared to the mean CSA of sham muscles (WT: 891 ± 110 *μ*m^2^, KO: 893 ± 167 *μ*m^2^). The CSA of regenerating fibers recovered to values similar to the sham muscles at 14 DPI in both genotypes (WT: 673 ± 120 *μ*m^2^, KO: 829 ± 154 *μ*m^2^ *P* = 0.09). No significant differences were found between genotypes for the recovery of muscle fiber CSA between genotypes (genotype × DPI × treatment, *P* = 0.52).

#### Embryonic myosin heavy chain

eMyHC is expressed in developing mammalian skeletal muscle in utero but is not normally present in most adult muscles. However, following injury, regenerating muscle fibers transiently express eMyHC and it thereby provides a useful marker of regenerating fibers (Schiaffino et al. 2015). Because we expected WT mice to show more effective muscle regeneration, we hypothesized that in the days following injury more muscle fibers of WT mice would express eMyHC compared to KO mice. CTX‐injected muscles had more eMyHC‐expressing fibers than sham‐injected muscles (treatment, *P* = 0.005) and muscles at 7 DPI had more eMyHC‐expressing fibers than at 14 DPI (DPI, *P* = 0.0009). However, no significant differences in eMyHC were found between genotypes (genotype *P* = 0.1, genotype × treatment *P* = 0.4, genotype × DPI × treatment, *P* = 0.3) at 7 DPI (WT: 56 ± 34.7 fibers, KO: 29 ± 8.4) or 14 DPI (WT: 4.4 ± 2.1 fibers, KO: 2.8 ± 3.4 fibers). These data are represented in Figure 3D, while Figure 3E shows representative images.

#### T‐cell immunostain

**Figure 4:**
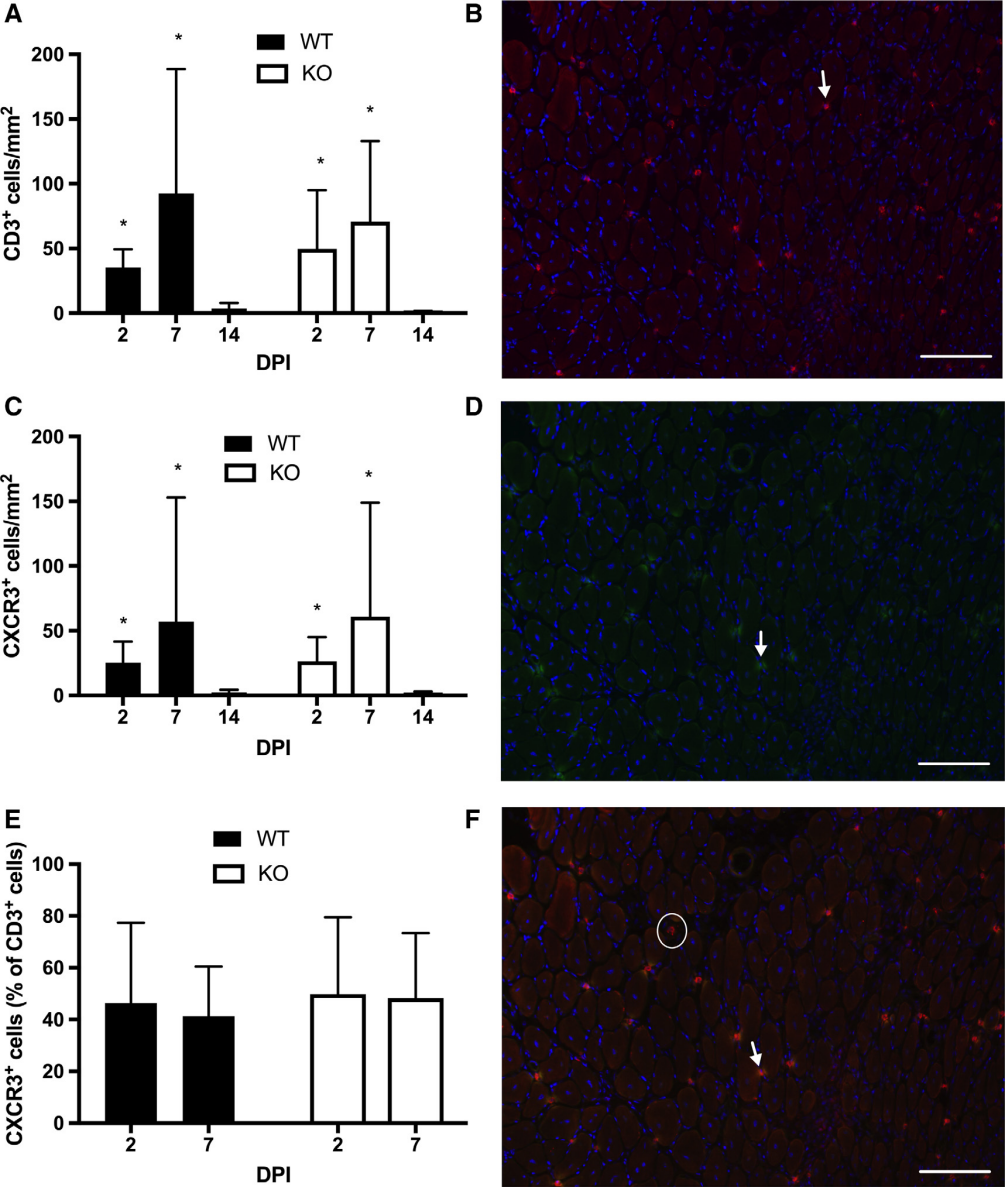
(A) T cells (CD3^+^ cells) were increased at 2 and 7 days postinjury (DPI) compared to 14 DPI (*, *P *< 0.05) in injured muscles of both wild‐type (WT) and CXCL10 knockout (KO) mice. (B) A representative micrograph of a T‐cell stain (Red = CD3, Blue = DNA) in a WT mouse at 7 DPI. Arrows point out examples of CD3‐positive cells. (C) CXCR3‐positive cells were increased at 2 and 7 DPI compared to 14 DPI (*P < 0.05) in both genotypes. (D) A representative micrograph of CXCR3 (green) stain in a WT mouse at 7 DPI. Arrows mark examples of CXCR3‐positive cells. (E) Approximately 50% of the T cells (CD3 cells) were CXCR3 positive (the CXCL10 receptor). No differences were observed between genotypes or between 2 and 7 DPI. (F) A merged image of panel B and D. The arrow points out an example a cell positive for both CD3 and CXCR3. The circle shows a CD3‐positive, CXCR3‐negative cell. Data are mean ± SD. Scale bar is 100 *μ*m. *n* = 4–6 per experimental condition.

To test whether the CXCL10/CXCR3 axis was necessary for T‐cell accumulation into muscle following toxin‐induced injury, we quantified the number of T cells (CD3^+^ cells) and cells bearing CXCR3 (CXCL10 receptor) in the muscles of WT and KO mice at 2, 7, and 14 DPI using immunohistochemistry. T cells were increased in both WT and KO muscle at 2 DPI (WT: 35 ± 14 cells/mm^2^, KO: 50 ± 45 ± cells/mm^2^) and 7 DPI (WT: 93 ± 96 cells/mm^2^, KO: 71 ± 62 cells/mm^2^) compared to 14 DPI (WT: 4 ± 4 cells/mm^2^, KO: 0.2 ± 0.4 cells/mm^2^). No significant differences were observed in the muscle T‐cell content between genotypes (genotype *P* = 0.8, genotype × DPI *P* = 0.4, Fig. 4A). Similarly, CXCR3‐positive cells were significantly increased at 2 DPI (WT: 25 ± 16 cells/mm^2^, KO: 26 ± 19 cells/mm^2^) and 7 DPI (WT: 57 ± 63 cells/mm^2^, KO: 61 ± 88 cells/mm^2^) compared to 14 DPI (WT: 2 ± 2 cells/mm^2^, KO: 1 ± 2 cells/mm^2^). No significant differences were observed between genotypes (genotype *P* = 0.7, genotype × DPI *P* = 0.4 Fig. 4C). To test if the KO mice failed to specifically recruit T cells that bore CXCR3 into the damaged muscle, we tested whether there was a difference between the percentage of T cells that expressed CXCR3 (100*(CD3^+^ cells plus CXCR3^+^ cells/CD3^+^ cells) at 2 and 7 DPI. No significant differences were found at 2 DPI (WT: 46 ± 31%, KO: 49 ± 29%) or 7 DPI (WT: 41 ± 19, CXCL10: 48 ± 25, Fig. 4E).

#### CXCR3 ligand expression

**Figure 5:**
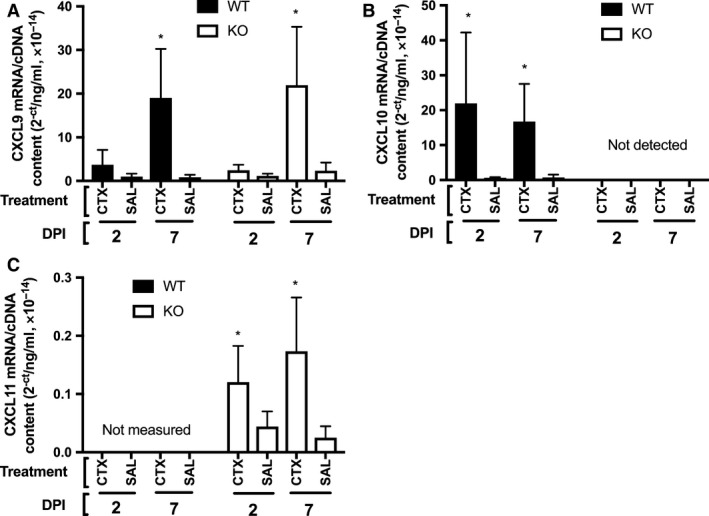
(A) CXCL9 gene expression was significantly increased in the injured muscles (CTX‐injected) compared to the uninjured muscles (saline‐injected, SAL) at 7 days post injury (DPI) in both wild‐type (WT) and CXCL10 knockout (KO) animals. (B) CXCL10 gene expression was significantly increased in the damaged muscles at 2 and 7 DPI in the WT animals. No CXCL10 mRNA was detected in the KO animals. (C) CXCL11 gene expression increased significantly in the knockout (KO) mice at both 2 and 7 DPI. * indicates significant difference from SAL (*P *< 0.05). Data are mean ± SD. *n* = 4–6 per experimental condition.

Because CXCL10 deficiency did not hinder muscle regeneration, we measured the gene expression of the other CXCR3 ligands in the damaged muscles to investigate the possibility that they compensated for CXCL10 in the KO mice. CXCL11 was not measured in the WT mice because they harbor a nullifying mutation in *cxcl11*. CXCL10 increased in the injured muscle relative to sham to a similar degree at both 2 and 7 DPI in the WT animals (treatment: *P* = 0.001, treatment × DPI: *P* = 0.96, Fig. 5B). As expected, no CXCL10 gene expression was detected in the KO animals (Fig. 5A). Relative to the sham muscle, CXCL9 expression in the injured muscle was increased at 7 DPI but not 2 DPI (time × treatment, *P* = 0.025, Fig. 5A). No differences in CXCL9 expression were found between genotypes (time × treatment × genotype, *P* = 0.6). CXCL11 expression in the KO mice increased significantly in the injured muscles relative to the sham muscles at 2 and 7 DPI (treatment: *P* = 0.003, treatment × DPI: *P* = 0.2, Fig. 5C).

## Discussion

Multiple immune cells, including macrophages, T cells, and eosinophils, play important roles in effective muscle regeneration (Tidball 2017). In parallel, cytokines and chemokines are also important in this process. Cytokines and chemokines have been found to support muscle regeneration directly by influencing satellite cell activity (Chazaud et al. 2003). They can also support muscle repair indirectly by recruiting (Lu et al. 2011b; Sun et al. 2009), or enriching (Kuswanto et al. 2016) needed immune cells in damaged muscle, or by influencing the phenotype of immune cells to carry out muscle regenerative functions (Arnold et al. 2015; Heredia et al. 2013). Some cytokines, such as IFN*γ* and TNF*α*, have been found to carry out needed functions in both the muscle parenchyma and immune cells in muscle regeneration (Tidball 2017). In two recent investigations (Deyhle et al. 2015; Hyldahl et al. 2014), we found CXCL10 was elevated in human muscle following muscle‐damaging lengthening contractions. To our knowledge, the role or necessity of CXCL10 in the regeneration of otherwise healthy muscle has not yet been investigated. We carried out this study to test whether CXCL10 was dispensable for effective muscle regeneration. The main finding of this study is that while CXCL10 is upregulated in human muscle following damage and it promotes myogenic differentiation in vitro*,* it appears to be dispensable for effective muscle regeneration.

CXCL10 is elevated in diseased muscle associated with chronic inflammation such as Duchenne muscular dystrophy (Villalta et al. 2009) and idiopathic inflammatory myopathies (Crescioli et al. 2012; De Paepe et al. 2005; Kim et al. 2014). Two recent reports from our laboratory were the first to report increases in CXCL10 in otherwise healthy muscle following contraction‐induced damage (Deyhle et al. 2015; Hyldahl et al. 2014). In both of these investigations, a 29‐plex cytokine assay was used. Thus, the possibility remained that this result was due to inflated risk of type I error associated with the multiplexing assay. Informed by these previous studies, we tested the a priori hypothesis that CXCL10 would be elevated in human muscle tissue following contraction‐induced injury. Here, we confirm that CXCL10 is indeed increased at both 24 and 72 h following muscle‐damaging contractions (Fig. 1C).

CXCL10 shares a common receptor (CXCR3) with the structurally similar chemokines CXCL9 and CXCL11 (Groom and Luster 2011). The signaling among these three ligands through CXCR3 is complex. While many functions are redundant, antagonistic functions have been identified among the CXCR3 ligands (Zohar et al. 2014). Other functions appear to depend preferentially on one ligand. For example, CXCL10 is essential for T‐cell‐mediated functions against *Toxoplasma gondii* infections (Khan et al. 2000). Due to the known complexity among the CXCR3 ligands, we measured CXCL9 and CXCL11 protein in the muscle biopsy samples to get a broader picture as to the potential CXCR3/ligand signaling axis following muscle damage. Neither CXCL9 nor CXCL11 changed significantly after damaging muscle contractions (Fig. 1C and D). This suggests that if any signaling through CXCR3 is important for muscle repair, CXCL10 appears to be the dominant ligand.

Other T_h_1 cytokines that are upregulated in regenerating muscle have been found to support muscle regeneration by influencing myogenic proliferation, differentiation, or fusion of muscle stem cells (Cheng et al. 2008; Warren et al. 2002). We found that CXCL10 did not impact proliferation of human primary myoblasts, but it did promote myogenic differentiation in vitro (Fig. 1F). These data suggest that CXCL10 might play a role in muscle regeneration directly, although myoblasts from this experiment were only derived from a single subject, making strong conclusions difficult to draw. Nevertheless, data from our in vivo loss of function study show that CXCL10 is dispensable for normal muscle regeneration following a myotoxic injury. In situ muscle strength was lost to the same degree and restored at the same rate in KO mice and WT mice. KO mice showed no histologically discernible deficiencies in regeneration compared to WT mice and KO mice showed no deficiency in nascent myofiber growth between 7 and 14 days.

The finding that a cytokine is upregulated in damaged/regenerating muscle and impacts myogenic cell activity yet is dispensable for normal muscle regeneration is not unprecedented. For instance, IL‐6 is upregulated in regenerating muscle (Tidball 2017; Warren et al. 2002) and it promotes satellite cell activity (Serrano et al. 2008). Yet, IL‐6 KO mice display normal muscle regeneration and force recovery following injury (Warren et al. 2002). It may be that CXCL10 is dispensable for muscle regeneration but serves important functions that require a more chronic stimulus to emerge. This is the case with IL‐6, as it is dispensable for muscle regeneration but it is needed for muscle hypertrophy following synergist ablation (Serrano et al. 2008).

Other chemokines, most notably CCL2 (also called MCP‐1), are important in the muscle regeneration process. Disrupting the CCL2/receptor (CCR2) axis leads to impaired muscle regeneration marked by fibrosis, adipose deposition, delayed function restoration, persistent necrotic tissue, and reduced nascent fiber growth (Contreras‐Shannon et al. 2007; Lu et al. 2011b; Warren et al. 2004). The muscle regeneration deficit in CCR2 KO mice, however, is largely rescued with a bone marrow transplant from a wild‐type donor mouse (Lu et al. 2011a; Sun et al. 2009). Thus, CCL2 appears to support muscle regeneration indirectly by recruiting monocytes to injured muscle (Lu et al. 2011a; Sun et al. 2009) and by influencing intramuscular monocyte/macrophage phenotype (Arnold et al. 2015). CXCL10 is a chemokine for T cells (Hoerning et al. 2011; Klein et al. 2005), which are vital participants in the muscle regeneration process (Burzyn et al. 2013; Fu et al. 2015; Kuswanto et al. 2016; Zhang et al. 2014). Because we recently found that CXCL10 and CD8+ T cells were significantly correlated in human muscle biopsy samples following muscle‐damaging contractions (Deyhle et al. 2015), we hypothesized that CXCL10 was needed for T‐cell recruitment to damaged muscle in a way similar to the relationship between CCL2 and monocytes/macrophages. To investigate this hypothesis, we stained for the pan T‐cell marker CD3. Contrary to our hypothesis, KO mice displayed similar muscle T‐cell accumulation at 2 and 7 DPI (Fig. 4A). Among the T cells present in the damaged muscle, about half were also positive for CXCR3 (Fig. 4C). Interestingly, a similar percentage of T cells expressed CXCR3 in muscle from human patients suffering from the inflammatory myopathy dermatomyositis (De Paepe et al. 2005).

Despite being upregulated in damaged muscle and influencing myogenic differentiation in vitro*,* CXCL10 is apparently not needed for effective muscle regeneration. The reason why KO mice did not exhibit any apparent muscle regeneration deficit may be that other CXCR3 ligands compensated for the absence of CXCL10. Studies show that the CXCR3 ligands can serve redundant functions. Some studies show that T‐cell trafficking is not impaired by the absence of one of the CXCR3 ligands (Campanella et al. 2008; Medoff et al. 2006). That CXCL9 gene expression was significantly elevated in both WT and KO mice (Fig. 5A), and that CXCR3+ T cells were also increased similarly between genotypes (Fig. 4E), lends credence to the possibility of CXCL9‐mediated compensation in the KO mice at 7 DPI. Other studies have shown that CXCL10 serves important nonredundant functions in specific immunological contexts. One study showed that CXCL10 KO mice failed to control neurotropic hepatitis virus infection in the brain, which was associated with reduced CD4 and CD8 T‐cell accumulation in the infected tissue (Dufour et al. 2002). The KO mice in that study failed to up regulate CXCL9 in the infected brain tissue. In contrast, KO mice in the present study successfully induced CXCL9 at 7 DPI (Fig. 5A). Thus, CXCL9 may have compensated for the absence of CXCL10 in the KO mice by recruiting T cells to the injured muscle tissue or by some other mechanism such as promoting myogenic differentiation. C57bl6 mice naturally harbor a severe mutation in the *Cxcl11 gene*. In these animals, a two‐nucleotide insertion shortly after the start codon causes a reading frame shift and premature stop codon (Sierro et al. 2007). Therefore, CXCL11 signaling in the WT animals is not possible. However, these data do not rule out the possibility of CXCL11‐mediated signaling in the KO animals. CXCL10 KO mice were developed using J1 embryonic stems that were backcrossed into the c57bl/6 line (Dufour et al. 2002). The use of J1 embryonic stem cells may have introduced an intact *cxcl11* gene into the KO mice. Due to this genetic discrepancy between the KO animals and their background strain, the KO mice might have compensated for the lack of CXCL10 by expressing CXCL11. Our data suggest that CXCL11‐mediated compensation is possible. CXCL11 expression did significantly increase relative to sham muscles at both 2 and 7 DPI (Fig. 5C). The level of CXCL11 expression is quite low: about 2 orders of magnitude lower than CXCL9 and CXCL10 at the respective time points (Fig. 5). However, the relatively low expression may be because among all of the CXCR3 ligands CXCL11 has the highest affinity for the receptor (Cole et al. 1998). As previously others have noted (Groom and Luster 2011), developing a CXCL10 and CXCL9 KO mice with C57BL/6 stem cells instead of J1 stem cells would allow researchers to identify the role of these chemokines without concerns of other genetic discrepancies.

## Conclusions

This investigation confirmed previous studies showing that CXCL10 is upregulated in human muscle following contraction‐induced damage. CXCL10 promoted myogenic differentiation of human primary myoblasts in vitro*,* suggesting possible direct involvement of CXCL10 in muscle regeneration. Yet, following a myotoxic injury, the absence of CXCL10 did not impair muscle regeneration as measured by muscle function, nascent fiber growth, and eMyHC expression. In addition, KO mice were not deficient in CXCR3^+^ T‐cell accumulation in injured muscle, suggesting this chemokine is also dispensable for T‐cell recruitment to injured muscle. More research is needed to determine whether other ligands might have compensated for the absence of CXCL10, and/or if CXCL10 might be needed for other more latent responses.

## Conflict of Interest

None declared.
